# Thrombocytopenia in the experimental leptospirosis of guinea pig is not related to disseminated intravascular coagulation

**DOI:** 10.1186/1471-2334-6-19

**Published:** 2006-02-02

**Authors:** Hong-Liang Yang, Xu-Cheng Jiang, Xiang-Yan Zhang, Wen-Jun LI, Bao-Yu HU, Guo-Ping Zhao, Xiao-Kui Guo

**Affiliations:** 1 Department of Pathology, Shanghai Jiao Tong University School of Medicine, Shanghai 200025, China; 2 Department of Microbiology and Parasitology, Shanghai Jiao Tong University School of Medicine, Shanghai 200025, China; 3 Chinese National Human Genome Center at Shanghai, Zhangjiang High Tech Park, Shanghai 201203, China

## Abstract

**Background:**

Thrombocytopenia is commonly observed in severe leptospirosis. However, previous studies on coagulation alterations during leptospirosis resulted in inconsistent conclusions. Some findings showed that the prominent levels of thrombocytopenia observed in severe leptospirosis did not reflect the occurrence of disseminated intravascular coagulation (DIC) syndrome, while the others reached the conclusion that the hemorrhages observed in leptospirosis were due to DIC. The aim of this study is to elucidate whether DIC is an important feature of leptospirosis.

**Methods:**

The leptospirosis model of guinea pig was established by intraperitoneal inoculation of *Leptospira interrogans *strain Lai. Hematoxylin and eosin (HE) staining, electron microscopy and immunohistochemistry staining were used to detect the pathologic changes. Platelet thrombus or fibrin thrombus was detected by HE, Martius Scarlet Blue (MSB) staining and electron microscopy. Hemostatic molecular markers such as 11-dehydrogenate thromboxane B2 (11-DH-TXB2), thrombomodulin (TM), thrombin-antithrombin III complex (TAT), D-Dimer and fibrin (ogen) degradation products (FDPs) in the plasma were examined by quantitative enzyme-linked immunosorbent assay (ELISA) to evaluate the hematological coagulative alterations in leptospirosis models.

**Results:**

Pulmonary hemorrhage appeared in the model guinea pig 24 hours after leptospires intraperitoneal inoculation, progressing to a peak at 96 hours after the infection. Leptospires were detected 24 hours post-inoculation in the liver, 48 hours in the lung and 72 hours in the kidney by immunohistochemistry staining. Spiral form of the bacteria was initially observed in the liver, lung and kidney suggestive of intact leptospires, granular form of leptospires was seen as the severity increased. Platelet aggregation in hepatic sinusoid as well as phagocytosis of erythrocytes and platelets by Kupffer cells were both observed. Neither platelet thrombus nor fibrin thrombus was found in the liver, lung or kidney via morphological observation. Thrombocytopenia was observed in all infected guinea pigs of our experimental leptospirosis study. Analysis of hematologic molecular markers showed that 11-DH-TXB2 and TM in the plasma were elevated significantly. TAT that reflects the thrombin activation had a trend of decline after infection. Although D-dimer and FDPs increased statistically, the increasing may not bear clinical significance.

**Conclusion:**

Pathologic and hematological studies for experimental leptospirosis of guinea pig indicated that the thrombocytopenia found in guinea pigs did not correlate with the occurrence of DIC. The platelet aggregation and Kupffer cells phagocytosis might be the potential causes of thrombocytopenia in severe leptospirosis.

## Background

Leptospirosis is a globally important zoonotic disease caused by pathogenic *Leptospira *species including *L. alexanderi, L. borgpetersenii*, *L. interrogans sensu stricto*, *L. kirschneri*, *L. noguchii*, *L. santarosai*, *L. weilii, L. fainei, L. inadai and L. meyeri*[1,2]. Human beings and animals become infected through contact with urine-contaminated soil and water. Leptospirosis is characterized by a broad spectrum of clinical manifestations, ranging from subclinical infection to Weil's syndrome, a severe and potentially fatal disease characterized by hemorrhage, acute renal failure and jaundice[3]. Although major progress has been made in the basic research of leptospirosis, the pathogenesis remains to be elucidated [4-6]. Haematological manifestations are common in leptospirosis and the most common finding in the severe cases is thrombocytopenia. However, its relationship with disseminated intravascular coagulation (DIC) remains controversial[7]. Some findings showed that although thrombocytopenia occurs in up to 50% of Weil's disease patients, the prominent levels of thrombocytopenia seen in severe leptospirosis often do not reflect the occurrence of DIC syndrome [8-12]. On the other hand, others reached the conclusion that the hemorrhages observed in leptospirosis are due to DIC [13-17]. The purpose of this study was to elucidate whether DIC is an important feature of experimental leptospirosis in guinea pig by pathologic and hematological studies.

## Methods

### Bacteria

The *L. interrogans *serogroup Icterohaemorrhagiae serovar lai type strain #56601 (strain Lai) was obtained from the Institute for Infectious Disease Control and Prevention (IIDC), Beijing, China. Leptospires were maintained by serial passages in guinea pigs for preservation of virulence and were cultured in liquid Ellinghausen-McCullough-Johnson-Harris (EMJH) medium at 28°C under aerobic conditions and collected at a density of about 10^8 ^bacteria per ml. The number of bacteria was manually counted with a Petroff Hausser counting chamber for experimental infection.

### Animals

The animal experiments were approved by the animal research committee of the Shanghai Second Medical University, and were conducted as described previously[12]. Briefly, forty guinea pigs of either sex were defined to five groups including the uninfected negative control. Each group contained eight guinea pigs, weighing 150 to 200 grams each. Guinea pigs were inoculated intraperitoneally with 1 ml of the leptospiral culture (5 × 10^8^). Eight normal guinea pigs were inoculated with EMJH medium as the negative controls. Guinea pigs were euthanized at 24, 48, 72 and 96 hours after infection.

### Pathologic studies

Tissues for histology studies were collected from normal and infected guinea pigs, fixed in neutral-buffered 4% formaldehyde, processed and stained for hematoxylin and eosin (HE), and Martius Scarlet Blue (MSB) according to routine procedures.

Tissues for ultrastructural studies were fixed in 2% glutaraldehyde, post-fixed in 1% osmium tetroxide, dehydrated in graded ethanols, and embedded in Epon 618. Ultrathin (70 nm) sections were stained with uranyl acetate and lead citrate, and examined with a PHILIP CM-120 electron microscope.

The rabbit antiserum specific to *L. interrogans *strain Lai was prepared using a modified procedure as previously described[18]. Immunohistochemistry staining was performed with the EnVision™ system, a highly sensitive two-step immunohistochemical technique[19], using commercially available immunohistochemistry kit (EnVision system, DAKO). In brief, paraffin-embedded tissue sections were dewaxed and rehydrated, treated with 3% H_2_O_2 _in methanol for 10 minutes, and then incubated in 1 mg/ml trypsin at 37°C for 15 minutes. Sections were incubated in primary rabbit antibody (1:6000 dilution) specific for *L. interrogans *strain Lai for 90 minutes, followed by EnVision™ for 40 minutes, visualized with DAB, and counter-stained with modified hematoxylin.

### Hematology

Blood sample was collected at 24, 48, 72 and 96 hours after infection by cardiac puncture immediately after anesthesia, and was anticoagulated with EDTA and citrate (final concentration, 3.2%). Platelet count was measured using an automated cell counter (Sysmex K-4500, Sysmex, Japan). The blood samples were centrifuged at 3000 rpm for 10 minutes. Plasma was aspirated and stored at -70°C for later analysis. Hemostatic molecular markers such as 11-dehydrogenate thromboxane B2 (11-DH-TXB2), thrombomodulin (TM), thrombin-antithrombin III complex (TAT), D-Dimer and fibrin(ogen) degradation products (FDPs) were measured with each respective commercial quantitative enzyme-linked immunosorbent assay (ELISA) kits (11-DH-TXB2: Cayman Chemical Company; TM: American Diagnostica Inc; TAT: DADE BEHING; D-dimer and FDPs: Sun Bioengineering Co. Ltd), as described) [20-22]. Briefly, according to the manufacturer's instruction, precoated 96-well microtiter plates were incubated with diluted plasma samples or serial dilutions of the respective standards, which were provided by the manufacturers. After color stabilization, the absorbance was measured by an automated ELISA-plate reader (KHB ST-360) with the suggested filters (420 nm for 11-DH-TXB2, 450 nm for TM, 492 nm for TAT, D-dimer and FDPs). The concentration of each sample was calculated in relation to the respective reference standard curve.

### Statistics

Hematologic results are presented as mean values ± SD. Statistical analysis was performed using analysis of variance (ANOVA). *P *< 0.05 was considered statistically significant.

## Results

### Macroscopic findings

**Figure 1:**
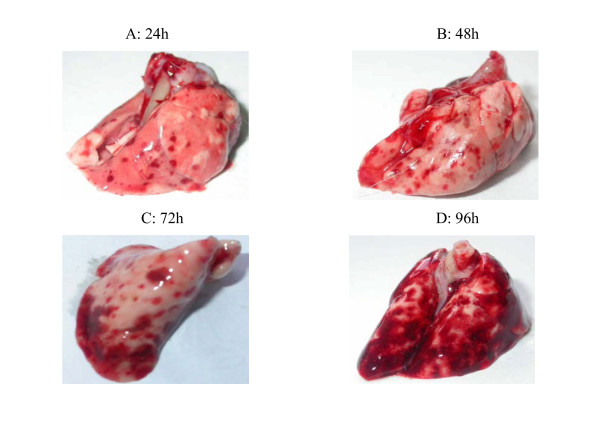
Hemorrhage of the lung of the guinea pigs injected with *L. interrogans*. A: 24 hours after infection; B: 48 hours after infection; C: 72 hours after infection; D: 96 hours after infection.

Distinct hemorrhage on the surface of the lung was observed 24 hours after infection with *L. interrogans *strain Lai. It became more severe, progressing to a peak at 96 hours. Meanwhile, diffused pulmonary hemorrhage was observed in 3 of 8 guinea pigs at the same stage (Fig 1). Generalized petechiae or ecchymoses in the peritoneum and other internal organs like heart, stomach, intestine and perinephros were also found.

### Microscopic studies

HE, MSB staining and ultrastructural studies showed that although there were conspicuous pathogenic changes in the liver, lung and kidney as described below, neither platelet thrombus nor fibrin thrombus was observed in these organs along with the course of leptospirosis.

### Liver

**Figure 2:**
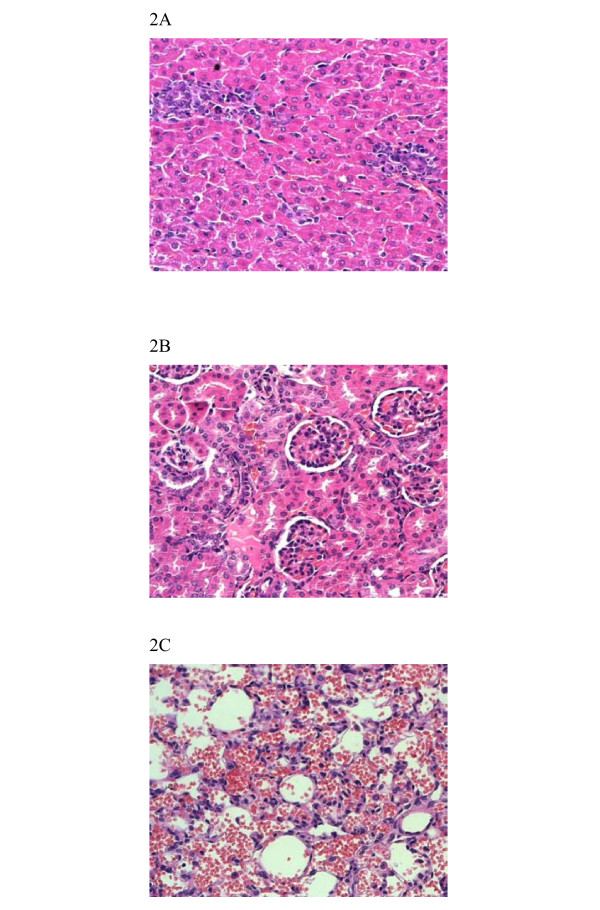
Pathologic changes in the liver (A), kidney (B) and lung (C) of guinea pigs infected with *L. interrogans*. (HE, magnification × 200).

Pathologic changes were first seen in the infected liver tissue at 48 hours after infection, progressing to the peak at 96 hours. Hepatocyte necrosis was observed either as groups or as scattered individual cells. Focal to diffuse cellular discohesion of hepatocytes was observed. Hypertrophy and hyperplasia of Kupffer cells were evident. Moderate increase of monocytes and neutrophils were observed in portal tracts (Fig 2A).

**Figure 3:**
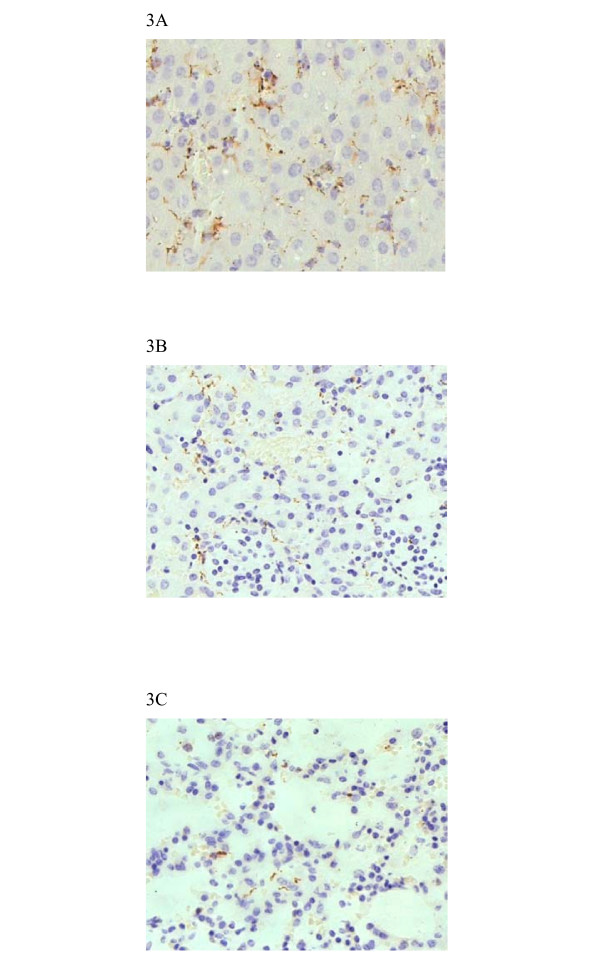
Positive immunohistochemistry staining in the liver (A), kidney (B) and lung (C) of guinea pigs infected with *L. interrogans*. (EnVision, magnification, × 200).

At 24 hours post-inoculation, spiral form of bacteria similar to intact leptospires began to appear in the liver by immunohistochemical staining. Starting from 48 hours after infection, leptospires were seen in granular forms (Fig 3A).

**Figure 4:**
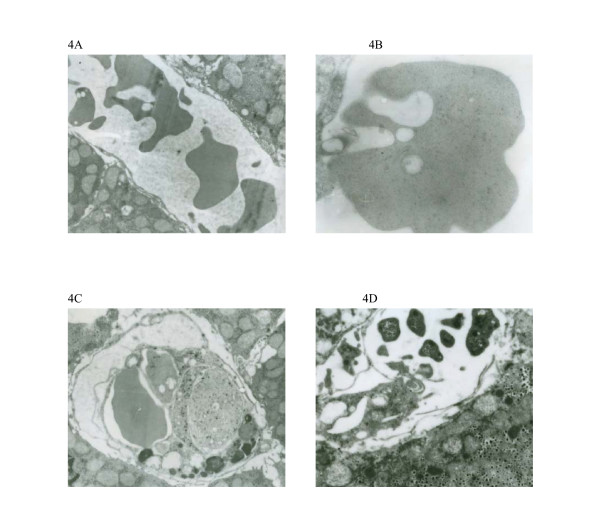
Electron photomicrographs of hepatic sinusoid of guinea pigs at 72–96 hours after infection. A: deformed erythrocytes (×4500); B: the breach of erythrocyte membrane (×12000); C: phagocytosis of erythrocytes and platelets by Kupffer cells (×4500); D: platelet aggregation (×3000).

Electron microscopy studies of liver at 72–96 hours after intraperitoneal inoculation showed that there were deformed erythrocytes in the hepatic sinusoid. Breaches were found in the membrane of erythrocytes. Platelet aggregation as well as phagocytosis of erythrocytes and platelets by Kupffer cells was observed (Fig 4A~D).

### Kidney

Interstitial nephritis, characterized by cellular infiltration composed of neutrophils and monocytes, was the major manifestation observed in the kidneys. Although no changes could be observed in the glomeruli, erythrocytes were seen in the lumen of renal tubules (Fig 2B).

Large number of intact leptospires was seen by immunohistochemical staining within the renal tubules, the interstitium, and the glomeruli at 72 hours after infection (Fig 3B). Degenerated granular leptospires were found in the interstitium and tubules at 96 hours after infection.

### Lung

Histological findings in the lung were consistent with that of the gross examinations. The alveolar hemorrhage appeared as small foci at 24 hours after infection, developed to coalesced areas of hemorrhage at 96 hours as severity increased. Slight-to-moderate inflammatory infiltrate and edema of the intra-alveolar septa were observed (Fig 2C).

Leptospires were detected inside septal capillaries and in interstitium at 48 hours after infection by immunohistochemistry staining. Leptospires in spiral form were initially observed. Starting from 72 hours after infection, leptospires were observed in granular forms. There were less leptospires in the lung as compared to the liver and kidney at the same stage (Fig 3C).

### Hematologic results

Hemotologic experiments including hemostatic molecular markers were analyzed along with the course of the disease to investigate the possible relationship between thrombocytopenia and DIC as shown in Table 1. Platelet counts of the infected guinea pigs decreased remarkably compare to the normal control (564 ± 82 × 10^9^/L) 24 hours after the infection (352 ± 40 × 10^9^/L), and this trend continued to 96 hours (30 ± 14 × 10^9^/L). Similarly, the mean platelet volume (MPV) increased in the same direction as that of platelet counts from 6.31 ± 0.19 fL of the normal control to 8.09 ± 0.33 fL at 96 hours after infection. In addition, the plasma level of 11-DH-TXB2, implying the activation of platelets, also increased after infection, progressing from 3.51 ± 0.55 μg/L in the control value to a peak of 7.46 ± 1.67 μg/L at 96 hours.

**Table 1:** Evolution of hematological detection in experimental leptospirosis in guinea pigs (*n *= 8 in each group), data are presented as *mean *± SD. (*P < 0.05, compare with control)

Characteristics	Control	24 h after infection	48 h after infection	72 h after infection	96 h after infection
Platelet count (10^9^/L)	564 ± 82	352 ± 40*	307 ± 50*	97 ± 35*	30 ± 14*
MPV (fL)	6.31 ± 0.19	6.53 ± 0.51	6.51 ± 0.18	7.04 ± 0.51*	8.09 ± 0.33*
11-DH-TXB2 (μg/L)	3.51 ± 0.55	5.12 ± 0.73*	4.85 ± 0.86*	5.31 ± 1.31*	7.46 ± 1.67*
TM (μg/L)	2.91 ± 0.30	3.40 ± 0.41*	5.63 ± 0.57*	6.62 ± 0.49*	6.87 ± 0.62*
TAT (μg/L)	3.66 ± 0.66	2.87 ± 1.01	3.27 ± 1.56	2.32 ± 0.77*	2.04 ± 0.25*
D-dimer (mg/L)	0.13 ± 0.01	0.19 ± 0.06*	0.24 ± 0.02*	0.27 ± 0.05*	0.19 ± 0.04*
FDPs (mg/L)	2.01 ± 0.08	1.99 ± 0.07	2.70 ± 0.53*	3.20 ± 0.43*	2.60 ± 0.56*

Plasma TM level, reflecting the status of endothelial cells damage, increased steadily, progressing from 2.91 ± 0.30 μg/L in the control value to a peak of 6.87 ± 0.62 μg/L at 96 hours after the infection.

The level of plasma TAT, reflecting the status of activating thrombin, showed a trend of decline after infection. It was 3.66 ± 0.66 μg/L in the control group but decreased to 2.04 ± 0.25 μg/L at 96 hours after the infection.

Plasma D-dimer and PDFs levels in experimental leptospirosis increased similarly comparing to the control group. D-dimer level in the control group was 0.13 ± 0.01 mg/L. It increased at 24 hours after the infection and progressed to a peak of 0.27 ± 0.05 mg/L at 72 hours. PDFs levels had a similar trend of increase from 2.01 ± 0.08 mg/L in the control group to a peak of 3.20 ± 0.43 mg/L at 72 hours after the infection.

## Discussion

Leptospirosis is a widely spread zoonotic disorder of global concern. It is characterized by a broad spectrum of clinical manifestations, ranging from subclinical infection or flu-like episodes to Weil's syndrome, a severe and potentially fatal disease with hemorrhage, acute renal failure (ARF) and jaundice[3]. In particular, massive pulmonary hemorrhages and fatal sudden haemoptysis may occur[23]. The complications of severe leptospirosis emphasize the multi-organ character of the disease. Thrombocytopenia is often observed in connection with hemorrhagic pneumopathy with septal capillary in severe human leptospirosis and is a significant predictor for the development of ARF[23]. However, the mechanistic relationship between thrombocytopenia and Weil's syndrome is yet to be established and its relationship with DIC, one of the common course of thrombocytopenia[24], remains controversial. DIC is characterized by systemic intravascular activation of the coagulation system, simultaneously leading to intravascular thrombi, and to bleeding as a consequence of the exhaustion of platelets and coagulation factors[25]. DIC is not a disease by itself and is always secondary to an underlying disorder, sepsis or severe infections induced by microbial pathogens[26].

The clinical presentation of DIC varies widely, which may complicate the clinical and laboratory diagnosis. Most of routinely available laboratory tests are capable of documenting a deficit in platelets and coagulation factors, but are not able to directly detect activation of coagulation. Since low levels of plasma coagulation factors may be caused by mechanisms other than DIC, many of the underlying conditions associated with DIC may cause a low platelet count in the absence of diagnosed DIC, such as the thrombocytopenia that may occur during severe infection. Global coagulation tests were not sensitive for pre-DIC, but molecular markers for activated platelets, fibrinolytic activity, vascular endothelial cell and activation of coagulation and fibrinogen to fibrin conversion are highly sensitive for the diagnosis of DIC. It is currently considered that hemostatic molecular markers such as TM, TAT, and D-dimer *et al *should be useful for early diagnosis of DIC[27].

Plasma 11-DH-TXB2 is a useful parameter of TXA2 formation, which implies the activation of platelets[28]. Plasma TM level is regarded as a molecular marker reflecting injury of endothelial cells. It is often increased in case of diffuse endothelial damage such as in DIC, diabetic microangiopathy, plasmodium falciparum and rickettsial infections. In several systemic inflammatory diseases, plasma TM levels are correlated to the activity of the disease[29]. Elevated plasma concentration of TAT may well reflect the increased generation of thrombin, and the high sensitivity may be helpful in detecting even low-grade activation of coagulation[30]. D-dimer is a specific fibrin degradation product and also serves as a marker for plasmin activation. FDPs, which serve as a marker of plasmin activation, are elevated in 85–100% patients with DIC, but the specificity of high levels of FDPs is limited and many other conditions, such as trauma, recent surgery, inflammation or venous thrombo-embolism, are associated with elevated FDPs. D-dimer levels are high in patients with DIC, but also poorly to distinguish patients with DIC from patients with venous thromboembolism, recent surgery or inflammatory conditions[31].

In the current study, we used pathologic and hematologic laboratory studies including hematologic molecular markers to elucidate whether DIC is an important feature of experimental leptospirosis in guinea pigs. Platelet aggregation and phagocytosis of platelets by Kupffer cells in hepatic sinusoid were observed. However, intravascular fibrin thrombi were absent in the kidney, liver or lung. These studies indicate that DIC is not a feature of experimental leptospirosis.

The hematologic laboratory results showed that thrombocytopenia was seen in the guinea pig models studied. Reduced platelet count with enlarged MPV suggesting that the thrombocytopenia was caused by accelerated platelet clearance rather than diminished production during *L. interrogans *infection. The increased plasma 11-DH-TXB2 levels indicated that there was platelet activation, followed by platelet aggregation and Kupffer cells phagocytosis. Increased TM level in guinea pig's plasma may reflect the injury of endothelial cells in leptospirosis. TAT that reflects the thrombin generation had a trend of decline after infection with *L. interrogans *other than increase. Although D-dimer and FDPs showed increase in our study, it may not be statistically significant for its clinical implication. In rats with lipopolysaccharide (LPS)-induced DIC, plasma concentrations of D-dimer and TAT were significantly increased to about one or two orders of magnitude comparing to the control group[32]. A few previous studies considered that thrombocytopenia in leptospirosis related to DIC. Those conclusions were based either on the occasional morphological observation of microthrombus in retrospective necropsy[16], or pathogical observation and routine laboratory tests related to coagulation factors such as prothrombin time, partial thromboplastin time and fibrinogen concentration in animal models of leptospirosis[13,17]. In contrary, Nally JE *et al *revealed that there was no chemical or microscopic evidence for DIC associated with thrombocytopenia for model guinea pigs, in that the D-dimer was not elevated, intravascular fibrin was not found[12]. Nicodemo AC *et al *concluded that thrombocytopenia, uremia and coagulation disorders, individually or as a group, should be included among the contributing factors that lead to and worsen bleeding episodes[33]. In our study, it is the first time that a group of highly sensitive hematologic molecular markers related to DIC, such as 11-DH-TXB2, TM, TAT, D-dimer and FDPs were used together to investigate the relationship of DIC in leptospirosis animal model. Our results indicate that, although the platelet activation and injury of endothelial cells in leptospirosis were revealed, the thrombocytopenia found in experimental leptospirosis did not correlate with the occurrence of DIC.

Thrombocytopenia can be a complication of many viral, bacterial, fungal, and protozoan infections. In some instances, infection-induced thrombocytopenia is severe enough to cause bleeding. Many, but not all, severe bacterial infections induce thrombocytopenia as a result of DIC, a condition precipitated by the generalized activation of the coagulation cascade in response to bacterial components such as LPS and peptidoglycan[34,35]. Although LPS of *L. interrogans *has a chemical structure and biologic effects similar to that of gram-negative bacteria, the former is approximately 12 to 20 times less toxic, and its role in the pathogenesis of the disease appears rather secondary[36,37]. Therefore, it is not surprising that the elevated levels of D-dimer and FDPs in the model guinea pigs may reflect the hematological toxicity of the leptospiral LPS, but not strong enough to cause any detectable DIC. It is highly likely that the platelet aggregation and Kupffer cells phagocytosis may be one of the potential causes of thrombocytopenia. The genome of *L. interrogans *encodes several proteins potentially related to haemostasis including bacterial collagenase, bacterial platelet-activating factor acetylhydrolase, von Willebrand factor type A domains-like protein and paraoxonase[38]. Further studies aiming at understanding the potential pathological effects of these and other related proteins such as hemolysin, might shed new lights on the mechanistic analysis of thrombocytopenia in leptospirosis.

## Conclusion

In summary, HE, MSB staining and ultrastructural studies showed that although there were conspicuous pathogenic changes in the liver, lung and kidney, neither platelet thrombus nor fibrin thrombus was observed in these organs along with the course of leptospirosis. Pathological studies indicate that DIC did not appear in experimental leptospirosis. The results of hematologic laboratory tests also indicate that although the platelet activation and injury of endothelial cells in leptospirosis were revealed, the thrombocytopenia found in experimental leptospirosis did not correlate with the occurrence of DIC. The platelet aggregation and Kupffer cells phagocytosis might be the potential causes of thrombocytopenia in severe leptospirosis.

## Competing interests

The author(s) declare that they have no competing interests.

## Authors' contributions

HLY, XCJ & XKG designed the research project. XCJ and WJL are the pathologists who examined tissue samples. XYZ and BYH coordinated the leptospira culture and participated in the pathology experiments. HLY carried out the hematology experiments. HLY, XKG and XCJ drafted the manuscript. GPZ participated in the design of the study and helped to draft the manuscript. All authors contributed in the writing and preparation of the manuscript. All authors read and approved manuscript.

## Pre-publication history

The pre-publication history for this paper can be accessed here:


